# CrERF5, an AP2/ERF Transcription Factor, Positively Regulates the Biosynthesis of Bisindole Alkaloids and Their Precursors in *Catharanthus roseus*

**DOI:** 10.3389/fpls.2019.00931

**Published:** 2019-07-18

**Authors:** Qifang Pan, Chenyi Wang, Zhiwei Xiong, Hang Wang, Xueqing Fu, Qian Shen, Bowen Peng, Yanan Ma, Xiaofen Sun, Kexuan Tang

**Affiliations:** ^1^Plant Biotechnology Research Center, Fudan-SJTU-Nottingham Plant Biotechnology R&D Center, School of Agriculture and Biology, Shanghai Jiao Tong University, Shanghai, China; ^2^Instrumental Analysis Center, Shanghai Jiao Tong University, Shanghai, China

**Keywords:** *Catharanthus roseus*, AP2/ERF transcription factor, bisindole alkaloids, transcriptional regulation, MIAs biosynthesis

## Abstract

*Catharanthus roseus* contains a variety of monoterpenoid indole alkaloids (MIAs), among which bisindole alkaloids vinblastine and vincristine are well-known to have antitumor effects and widely used in clinical treatment. However, their contents in *C. roseus* is extremely low and difficult to meet market demands. Therefore, it is of great significance to study the transcriptional regulation mechanism of MIAs biosynthesis for high yielding of bisindole alkaloids in *C. roseus*. Studies have shown that MIAs biosynthesis in *C. roseus* has complex temporal and spacial specificity and is under tight transcriptional regulation, especially bisindole alkaloids. In this study, an AP2/ERF transcription factor CrERF5 was selected by RNA-seq of *C. roseus* organs, and its full-length sequence was cloned and characterized. CrERF5 responds to both ethylene and JA signals and is localized in the nucleus. CrERF5 could activate the transcriptional activity of the TDC promoter. Transient overexpressing CrERF5 in *C. roseus* petals caused a significant increase of the expression levels of key genes in both the upstream and downstream pathways of MIAs biosynthesis while silencing *CrERF5* resulted in a decrease of them. Accordingly, the contents of bisindole alkaloids anhydrovinblastine and vinblastine, monoindole alkaloids ajmalicine, vindoline, and catharanthine were strongly enhanced in *CrERF5*-overexpressing petals while their contents decreased in *CrERF5*-silenced plants. These results suggested that CrERF5 is a novel positive ethylene-JA-inducible AP2/ERF transcription factor upregulating the MIAs biosynthetic pathway leading to the bisindole alkaloids accumulation.

## Introduction

*Catharanthus roseus* produces a wide range of bioactive monoterpenoid indole alkaloids (MIAs), which could be divided into two categories: monoindole alkaloids and bisindole alkaloids (Pan et al., 2016a). Both have high economic values due to their wide spectrum of pharmaceutical applications, among which bisindole alkaloids vinblastine and vincristine are the best-known antitumor compounds of significant clinical medical values. The first MIA strictosidine is formed by the coupling of tryptamine and secologanin, products of the indole and iridoid pathways, respectively. In recent years, key biosynthetic enzymes in the steps from strictosidine to ajmalicine, vindoline, and catharanthine have been identified and characterized. Strictosidine is deglucosylated by Strictosidine β-glucosidase (SGD) to form a highly reactive aglycone, which can be converted to ajmalicine by Heteroyohimbine Synthase (HYS), or to stemmadenine by Geissoschizine Synthase 1 and 2 (GS1 and 2) in conjunction with Geissoschizine Oxidase (GO) and the recently published Redox1 and Redox2 (Tatsis et al., 2017; Qu et al., 2018). Next, stemmadenine is acetylated by Stemmadenine-O-Acetyltransferase (SAT) to form O-acetylstemmadenine, which is further converted to catharanthine by α/β Hydrolase 1 (HL1) or to tabersonine by α/β Hydrolase 2 (HL2) (Qu et al., 2018). And tabersonine is converted into vindoline by the action of seven enzymes (Yang et al., 2015). Finally, the monoindole alkaloids vindoline and catharanthine are coupled by CrPRX1 to produce bisindole alkaloids anhydrovinblastine, the direct precursor of vinblastine and vincristine (Verpoorte et al., 1997; Costa et al., 2008).

Monoterpenoid indole alkaloid biosynthesis in **C. roseus** is an organ and cell-type specific pathway under tight transcriptional regulation (Courdavault et al., 2014; Thomas et al., 2015). Up to now, six types of transcription factor (TF) genes involved in the MIA pathways have been cloned and identified. AP2/ERF TFs, ORCAs (octadecanoid-responsive Catharanthus AP2-domain proteins), are firstly identified as the major regulators of several MIA pathway genes in **C. roseus** (Menke et al., 1999; Van der Fits and Memelink, 2000). ORCA3 has been reported to have the ability to upregulate the expression of several MIAs biosynthetic genes, like **anthranilate synthase (AS), tryptophan decarboxylase (TDC), D-1-deoxyxylulose 5-phosphate synthase (DXS), cytochrome P450 reductase (CPR), strictosidine synthase (STR)*,*and **desacetoxyvindoline 4-hydroxylase (D4H)** but not *SGD*, *deacetylvindoline 4-O-acetyltransferase (DAT)*, **CrPRX1** and the iridoid pathway genes (Van der Fits and Memelink, 2000). ORCA4 and 5 form a physical cluster and functionally overlap with ORCA3 (Paul et al., 2017). CrMYC2 in bHLH (basic helix-loop-helix) family could regulate the expression of ORCAs and also had a strong effect on the accumulation of catharanthine and tabersonine (Montiel et al., 2011; Zhang et al., 2011). CrMYC2 and ORCA3 have both been shown to be capable of transactivating the **TDC** promoter in tobacco protoplasts (Paul et al., 2017), but no report of transactivation on the downstream genes promoters of MIA pathway. Another two bHLH TFs, BIS1 and BIS2 act in a complementary manner to ORCAs and transactivated the expression of all genes in the iridoid pathway (Van Moerkercke et al., 2015). CrWRKY1 possesses overlapping functions with the ORCA TFs. Similar to ORCA3, CrWRKY1 activates pathway enzymes including AS, DXS, SLS1, SGD, and TDC (Suttipanta et al., 2011). Negative regulators, like CR1 from AP2/ERF family, RMT1 from bHLH family, zinc finger-binding proteins (ZCTs), CrGBF1, and CrGBF2 from bZIP family, are characterized to repress the transcription of **CrMYC2, ORCAs, STR*,* and **TDC** (Memelink et al., 2001; Sibéril et al., 2001; Chatel et al., 2003; Pauw et al., 2004; Zhang et al., 2011; Patra et al., 2013, 2017; Thamm et al., 2016; Zhou and Memelink, 2016; Liu et al., 2017). Besides, MYB-like transcription-factor CrBPF1 was found binding to an enhancer domain of the **STR** promoter (Van der Fits et al., 2000).

Positive and negative regulatory loops consisting of these activating and repressing TFs operate in the MIA pathway. Upon JA induction, active CrMYC2 released by JAZ proteins induces expression of BIS and ORCA TFs, forming positive auto-or cross-amplification loops for the MIA biosynthesis genes. In parallel, active CrMYC2 is supposed to induce transcription of **JAZ**repressors, leading to a negative regulatory loop involving BIS1 and RMT1, which maintains a balance of MIA precursor flux and also limits the increase of MIAs accumulation (Patra et al., 2017; Paul et al., 2017; Schweizer et al., 2018). What’s more, the primary regulatory loop of CrMYC2, BIS1/2, and ORCAs has limited effect on the accumulation of bisindole alkaloids (Schweizer et al., 2018). Overexpressing either **ORCA2** or **ORCA3** in **C. roseus** suspension cells or hairy roots enhanced the contents of tryptamine, catharanthine, serpentine, and ajmalicine (Van der Fits and Memelink, 2000; Peebles et al., 2009; Li et al., 2013; Sun and Peebles, 2016). Overexpressing **ORCA4** in hairy roots increased the accumulation of tabersonine, catharanthine, and ajmalicine (Paul et al., 2017). Bisindole alkaloids (like anhydrovinblastine, vinblastine, and vincristine) were below the detection levels even when overexpressing **ORCAs** in **C. roseus** cell cultures and hairy roots (Wang et al., 2010; Li et al., 2013). In **C. roseus** plants **ORCA3** overexpression significantly increased the accumulation of strictosidine, vindoline, catharanthine, and ajmalicine but failed to affect the anhydrovinblastine and vinblastine levels (Pan et al., 2012). Negative AP2/ERF TF CR1 only involved in the accumulation of vindoline and serpentine in **C. roseus** (Liu et al., 2017). It was reported that an AP2/ERF TF might interact with CrPRX1 to play a key role in the vinblastine content (Wang X. et al., 2016). But no further investigation was performed. These researches strongly suggest that there should be undiscovered AP2/ERF TFs participating in the regulation of bisindole alkaloids.

In our study, combined with transcriptome sequencing data, a newly AP2/ERF TF, CrERF5, was selected as a potential regulator on the MIA pathway leading to the accumulation of binsindole alkaloids. Petal transient expression system and virus-induced gene silencing (VIGS), two efficient and effective screening platforms for gene discovery and assessment, were used to test the gene function of CrERF5, which provided experimental evidence that CrERF5 plays a positive role in regulating the expression of several key genes in the upstream and downstream pathways of MIA biosynthesis and boosts the bisindole alkaloids accumulation.

## Materials and Methods

### Plant Growth and Treatments

Seeds of **C. roseus** (cv. Vitae Rose Red) were purchased from Floranova Ltd. (Dereham Norfolk NR20 4SS United Kingdom). The seeds were surface sterilized in 75% (v/v) ethanol for 1 min and 10% (v/v) NaClO_2_ for another 10 min. Subsequently, seeds were washed 10 times with sterile distilled water and germinated on Petri dishes containing MS basal medium. Cultures were grown under 16 h light and 8 h dark photoperiod at 25 ± 2°C. After germination for 2 weeks, seedlings were transplanted into soil and grown in the greenhouse at 25 ± 2°C. Flowers, leaves, stems, and roots of at least three biological replicates were collected in September 2018 during the blooming season of **C. roseus**. Two types of leaf samples were collected according to their position in the plants: younger leaves from the top four upper layers and older leaves from the bottom four lower layers.

MeJA and ethephon (ETH) were purchased from Sigma-Aldrich. The stocks were prepared by dissolving them in ethanol to make a concentration of 1 M. The working solution of MeJA and ETH was 0.1 mM (diluted by water) and used for the treatments. The sterile water was used as mock treatment for the controls. After spraying the working solutions, *C. roseus* leaves were collected at 0, 3, 6, 12, and 24 h. The experiments were conducted in randomized block design (RBD) with three replications.

### Analysis of the RNA-Seq Data

The transcriptome sequence of *C. roseus* retrieved from the NCBI was used as the reference database (Góngora-Castillo et al., 2012). The RNA-seq data of different tissues (SRR122239 for flowers; SRR122251 for leaves; SRR122253 for stems; SRR122254 for roots) were downloaded from the NCBI. Transcript abundant estimation and expression level in fragment per kilobase exon per million mapped fragment (FPKM) were performed as described by Wang A. et al., 2016 (Supplementary Table S2). AP2/ERF TFs were identified in the *C. roseus* transcriptome sequences using BLASTP queries with the known *Arabidopsis* AP2/ERFs. Hierarchical cluster (HCL) analysis was performed using a Multi Experiment Viewer (MEV, v.4.9.0) (Howe et al., 2010). Sample clustering was carried out using the HCL method and the evolutionary distances were computed with the Pearson correction and average linkage clustering (default parameters). The maximum FPKM in the color scale of the HCL heatmap was set up as 100. The putative AP2/ERF transcription factor sequences of *C. roseus* and five other plant species were aligned by CLUSTAL X (Trinity College, Dublin, Ireland). The phylogenetic tree was constructed by MEGA 5 software (Kumar et al., 2016). Protein sequences of AP2/ERF transcription factors (TFs) from other species were also gathered using the NCBI.

### Cloning of *CrERF5* Gene

The full-length ORF of CrERF5 was PCR-amplified from the leaf cDNA of *C. roseus* (primers shown in Supplementary Table S1). The PCR product was gel-purified (Axygen, United States), and cloned into the pLB-Simple vector (MIAngen, Shanghai, China) for sequencing (Sangon Biotech, Shanghai, China) and expression constructs generation.

### Subcellular Localization

To analyze the subcellular localization, the full-length ORF of CrERF5 was cloned into BamHI and XbaI sites of the pHB-YFP expression vector (Zhong et al., 2018). The construct was introduced into Agrobacterium tumefaciens strain GV3101, which was transiently transformed into 5-week-old *Nicotiana benthamiana* leaves following the published protocol (Voinnet et al., 2003; Yan et al., 2016). The YFP signal was observed after 48–72 h cultivation in low-light conditions using confocal microscopy (Leica Microsystems, Wetzlar, Germany), with argon laser excitation at 514 nm and a 505–550 nm emission filter set.

### Dual-Luciferase (Dual-LUC) Assay

The assay was performed as previously reported (Shan et al., 2014). P2300-CrERF5 was transformed into *A. tumefaciens* strain GV3101 to act as effectors. PGREEN-TDC-pro, PGREEN-SGD-pro, PGREEN-DXS1-pro, and PGREEN-CrPRX1-pro were separately fused to the luciferase and subsequently co-transformed with the helper plasmid pSoup19 into GV3101 to act as the reporter. The P2300 empty vector (EV) was used as a negative control. The 35S promoter-driven Renilla was used as the internal reference. After 48 h cultivation in low-light conditions, the leaf samples were collected for Dual-LUC assay using commercial Dual-LUC reaction reagents (Promega). Six biological repeats were measured for each sample.

### Virus-Induced Gene Silencing

Seedlings of *C. roseus* with two to three pairs of true leaves were used for VIGS. The genomic DNA of *C. roseus* was used as template to amplify the partial fragment of CrERF5 (386 bp) with specific primers (Supplementary Table S1). The PCR-generated fragment was then introduced into the terminal vector pTRV2 by Gateway LR Clonase^*TM*^ II (Invitrogen, Thermo Fisher Scientific, United States). *Catharanthus Protoporphyrin IX magnesium chelatase subunit H* (*ChlH*) was selected as a useful marker for determining the best time to collect samples for testing (Liscombe and O’Connor, 2011). The purified plasmid pTRV2-CrERF5, pTRV2-EV, pTRV2-*ChlH* were respectively transformed into *A. tumefaciens* strain GV3101. Three kinds of strains and GV3101 harboring pTRV1 were cultured overnight at 28°C in 30 mL Luria-Bertani medium containing 10 Mm MES, 20 μM acetosyringone, and 50 μg/mL kanamycin. These bacterial pellets were collected and then resuspended in 10 mL infiltration buffer (10 mM MES, 200 μM acetosyringone, and 1 mM MgCl_2_), and further incubated at 28°C for 3 h with shaking. The suspension of strain harboring pTRV1 was mixed with pTRV2-CrERF5, pTRV2, or pTRV2-*ChlH* in equal volume just before the infection. Young plants were wounded using toothpicks through the stem just below the apical meristem, and infiltrated with sterile water (mock inoculation), with the mixture of *A. tumefaciens* cultures harboring pTRV2-EV/pTRV1, pTRV2-CrERF5/pTRV1, and pTRV2-*ChlH*/pTRV1, respectively. Each treatment was injected into nine plants. After injection, the plants were cultured in a constant temperature incubator. The *ChlH* phenotype was observed 3 weeks after inoculation, and the other three sample plants were harvested at this stage. Leaves from three different plants per sample were cut in two. One sample was used for RNA extraction for gene expression analysis and the other sample was used for metabolites analysis. After recording the fresh weights of harvested materials, the samples were frozen in liquid nitrogen. Three replicates were made for each sample.

## Transient Transformation in *Catharanthus Roseus* Petals

The p2300-CrERF5 construct was made for *Agrobacterium*-mediated transient transformation in *C. roseus* petals. Flower petals of 1-year-old *C. roseus* plants (grown under greenhouse conditions) were infiltrated with *A. tumefaciens* GV3101 harboring the respective constructs p2300-p*CaMV35S:CrERF5* and p2300-p*CaMV35S:GUS* (as a control vector) for overexpression as previously described (Schweizer et al., 2018). After 48 h of incubation with *A. tumefaciens* solution, petals from five different flowers per sample were cut in two and immediately flash-frozen in liquid nitrogen. One sample was used for RNA extraction for gene expression analysis and the other sample was used for metabolite profiling. Three replicates were made for each sample.

### Relative Expression Analysis via Quantitative RT-PCR

For quantitative RT-PCR (qRT-PCR), total RNA was isolated from the leaves stored at −20°C. DNA contamination was removed using DNase I following the protocol provided by the manufacturer (TaKaRa, Japan). The cDNAs were synthesized from the RNA samples using Prime Script^*TM*^ Reverse Transcriptase Reagent according to the manufacturer’s instructions, using oligo (dT) as primer. The qRT-PCR analysis was performed in Peltier Thermal Cycler PTC200 (Bio-Rad), using the cDNAs as templates and gene-specific primers (in Supplementary Table S1) for the gene analysis. The primers for the MIA biosynthetic genes are listed in Supplementary Table S1 (Schweizer et al., 2018). SYBR Green (SYBR Premix Ex Taq; TaKaRa) was used in the PCR reactions to quantify the amount of dsDNA. The relative Ct (threshold cycle value) method (User Bulletin 2, ABIPRISM700 Sequence Detection System, update 2001; PerkinElmer/Applied Biosystems) was used to estimate the initial amount of template present in the reactions.

### Metabolite Analysis by UPLC-Q/TOF MS

UPLC-Q/TOF MS analyses were performed using a Primer UPLC-Q-TOF mass spectrometer (Waters Corp., Milford, MA, United States) equipped with an electrospray ionization source. Data acquisition, handling and instrument control were performed using MassLynx 4.1 software. Mass range, m/z 50–1000 in positive mode; capillary, 3.0 kV; sample cone voltage, 35 V; extraction cone, 3.0; ion guide, 3.0; source temperature, 115°C; desolvation gas temperature, 300°C; flow rate of desolvation gas, 700 L h^–1^.

To ensure accuracy and reproducibility, all analyses were conducted using an independent reference spray via the Lock Spray interface; Tyr–Gly–Gly–Phe–Leu (leucine-encephalin, 200 pg μL^–1^) was used as a lock mass (m/z 556.2771) under positive-ion conditions for real-time calibration (flow rate of 30 μL min^–1^). Before the experiment, a single-point calibration was performed against the lock mass compound (leucine-encephalin). A multiple-point calibration was then performed over the range m/z 50–1000 using sodium formate solution (prepared from 10% formic acid/0.1 mol L^–1^ sodium hydroxide solution/acetonitrile, 10 mL/10 mL/80 mL). All points fell within 1 ppm during calibration. The resolving power of the instrument was 8000.

UPLC conditions: a BEH C18 (100 mm × 2.1 mm, 1.7 μm) column (Waters) was used; the column temperature was maintained at 40°C. Mobile phases A (water, 0.1% formic acid) and B (acetonitrile, 0.1% formic acid) were used; the gradient program was as follows: 0–4 min 5–25% B, 9–12 min 45–85% B, 14 min 100% B; 14.5–16 min 5% B, flow rate 0.35 mL min^–1^; injection volume 2 μL. Acquity PDA detector wavelength was fixed at 210, 254, and 278 nm.

A mixture of reference standards of MIAs and precursors (secologanin, ajmalicine, catharanthine, and vinblastine were purchased from Sigma-Aldrich, St. Louis, MO, United States; vindoline and anhydrovinblastine were purchased from Shanghai R&D Center for Standardization of Chinese Medicines, China) were detected and identified based on MS/MS spectra (Supplementary Figures S1, S2). Samples were applied in triplicate for quantification using calibration curves of the standards.

### Data Statistical Analysis

All experiments were conducted with three replicates. Statistical analysis was performed using the student’s *t*-test by SPSS (version 14.0, Chicago, IL, United States). The values are mean ± SD for replicates in each group. *p* values ≤ 0.05 were considered as significant.

## Results

### An AP2/ERF TF Was Selected by RNA-Seq

**FIGURE 1:**
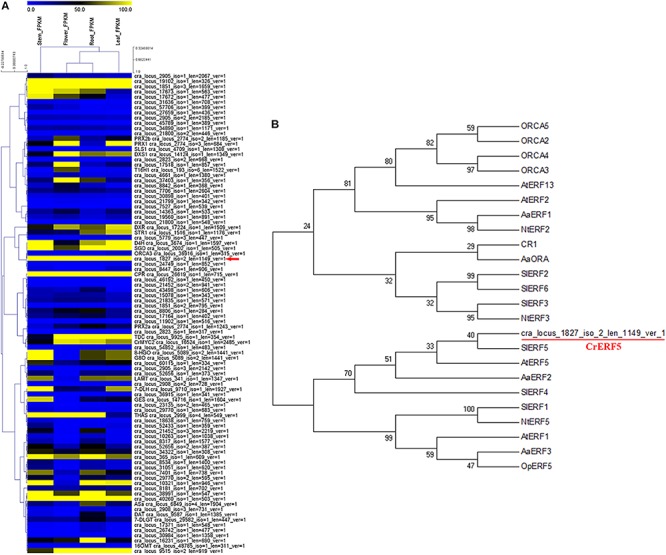
Identification and sequence analysis of CrERF5. **(A)** Hierarchical cluster analysis of AP2/ERF transcription factors in *C. roseus* flower, leaf, stem, and root. The average linkage hierarchical clustering with Pearson correlation was used. The color scale at the top represents the value of transformed reads per kilobase per million mapped reads. **(B)** Phylogenetic analysis of CrERF5 and other known AP2/ERF TFs from five plant species. *Arabidopsis* (At)*, Solanum lycopersicum* (Sl)*, Artemisia annua* (Aa), *Nicotiana tabacum* (Nt), and *Ophiorrhiza pumila* (Op). The tree presented here is a neighbor-joining tree based on amino acid sequence alignment.

To investigate more MIA biosynthesis-related AP2/ERF TFs, we screened the transcriptome database of *C. roseus* tissues and identified 69 AP2/ERF proteins by performing a BLASTP analysis with *Arabidopsis* AP2/ERF proteins. Hierarchical cluster analysis of 69 *C. roseus* AP2/ERF TFs and key regulatory and biosynthetic genes in the MIAs pathway revealed one AP2/ERF TF gene (ID: cra_locus_1827_iso=2_len=1147_ver=1) that was found to be clustered with SGD, ORCA3, and D4H in the heatmap (Figure 1A).

Phylogenetic analysis was performed to investigate the evolutionary relationships between the putative AP2/ERF TF and 22 AP2/ERF proteins from five other plant species (*Arabidopsis, Solanum lycopersicum, Artemisia annua, Nicotiana tabacum, Ophiorrhiza pumila*). The putative AP2/ERF TF was classified to the same group with SlERF5, AtERF5, NtERF5, SlERF4, and AaERF2, and showed the closest relationship with SlERF5 (Figure 1B). Therefore, the candidate AP2/ERF TF was designated as CrERF5 for further functional study. CrERF5 encoded 342 amino acids and its predicted protein consisted of an AP2/ERF domain with 60 amino acids (177–227) in the central region (Supplementary Figure S3).

### Subcellular Localization and Expression Pattern of CrERF5 Protein

**FIGURE 2:**
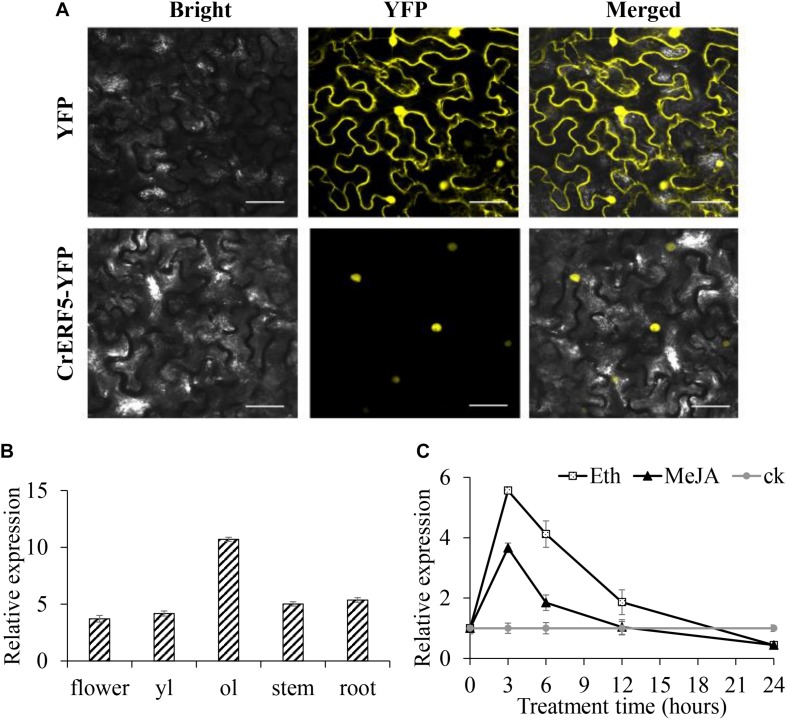
Subcellular localization and expression of CrERF5. **(A)** Subcellular localization of CrERF5 in tobacco leaves. Bars = 30 μm. **(B)** Relative expression of *CrERF5* in flower, younger leaf (yl), older leaf (ol), stem, and root of *C. roseus*. **(C)** The expression pattern of *CrERF5* in *C. roseus* leaves after treatment with 0.1 mM MeJA, 0.1 mM ETH, and sterile water (ck). The values of hormone treated samples were normalized to the ck samples at each time point, and the ck samples were normalized to themselves. The *C. roseus* N2227 gene was used as an internal control. The error bars represent the means ± SD (standard deviation) from three replicates.

To investigate the subcellular localization of CrERF5, laser scanning confocal microscopy was used to detect the YFP fluorescence signal of the fusion protein CrERF5-YFP expressed in *N. benthamiana* leaves. As illustrated in Figure 2A and Supplementary Figure S4, the YFP signal is specifically localized in the nucleus, consistent with CrERF5-YFP functioning as a transcription factor.

The expression pattern of *CrERF5* in the various tissues of *C. roseus* was analyzed through qRT-PCR. The results revealed that *CrERF5* showed the highest expression level in the older leaves, and had a higher expression level in the flowers and stems than in the younger leaves and roots (Figure 2B). To further investigate the expression characteristics of CrERF5, we examined the time-course expression of *CrERF5* under MeJA and ETH hormone treatments. As shown in Figure 2C, *CrERF5* expression increased 5.6-fold over the control at 3 h and then back to the normal level during 12–24 h after ETH treatment. Under MeJA treatment *CrERF5* showed a similar expression pattern with an increase of 3.7-fold over the control at 3 h. The results suggested that CrERF5 is a regulator that responds to both ethylene and JA signal.

### Transactivation Activity of CrERF5 on MIA Biosynthetic Genes

**FIGURE 3:**
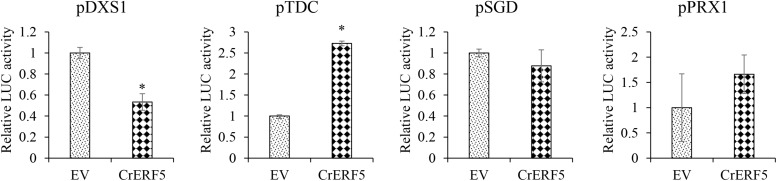
Transient Dual-luciferase assay of CrERF5 with MIA pathway genes (*TDC, DXS1, SGD*, and *CrPRX1*) promoters in tobacco. The relative LUC activity was normalized to the reference renilla (REN) luciferase. The error bars represent the means ± SD from three biological replicates, and asterisks indicate statistically significant differences compared with the controls. ^*^P < 0.05.

In order to experimentally verify the involvement of CrERF5 in MIA pathway, Dual-LUC assay was used to determine the transactivation ability of CrERF5. Given the co-expression analysis, the SGD, DXS1, TDC, and CrPRX1 promoters were employed in the Dual-LUC assay (Figure 3). The results showed that CrERF5 significantly activated the TDC promoter by 2.7-fold compared to the controls, but slightly activate the CrPRX1 promoter and did not activate the SGD promoter. Furthermore, CrERF5 significantly repressed the transactivation of DXS1 promoter, which was not co-expressed with CrERF5.

### Virus-Induced Gene Silence of CrERF5 Repressed the MIA Biosynthetic Pathway

**FIGURE 4:**
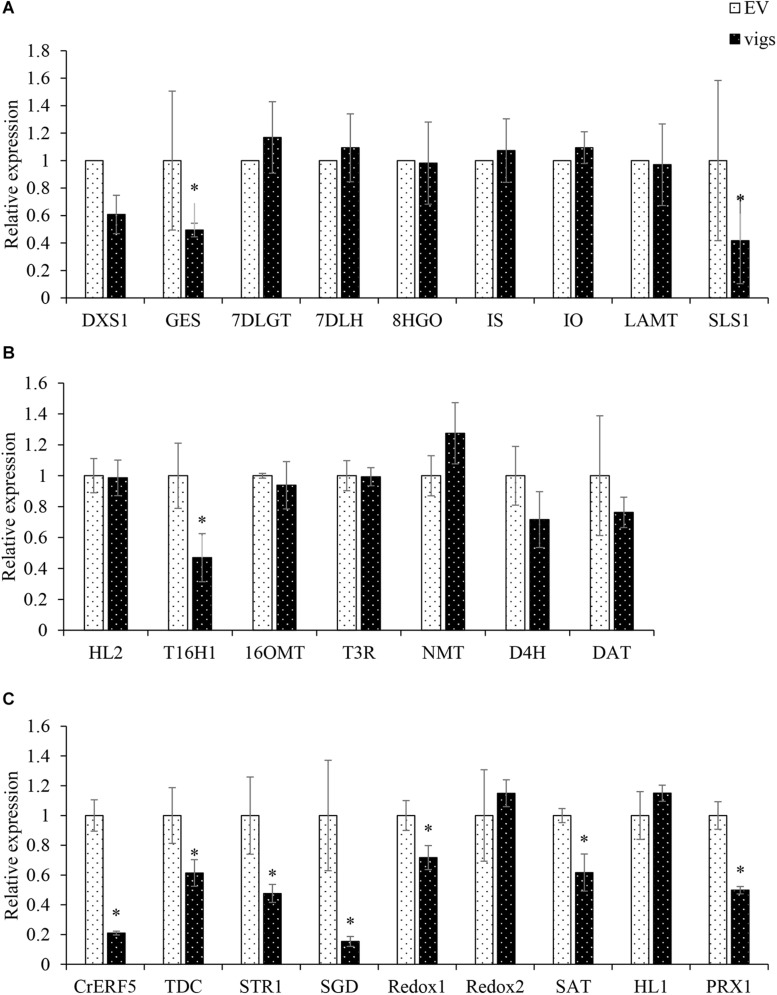
Expression of *CrERF5* and MIA pathway genes in *CrERF5*-silencing *C. roseus* lines. **(A)** Iridoid pathway genes; **(B)** Vindoline pathway genes; **(C)** Indole and MIA pathway genes. The error bars represent the means ± SD from three biological replicates, and asterisks indicate statistically significant differences compared with the controls. ^*^P < 0.05.

In order to identify the gene function of CrERF5 in MIA pathway, VIGS was used to silence the expression of *CrERF5* in *C. roseus* leaves. Transcripts analysis of *CrERF5* and other 25 genes in the indole pathway, iridoid pathway, vindoline pathway and MIA biosynthetic pathway were monitored in *C. roseus* leaves by qRT-PCR. *CrERF5* expression was suppressed by approximately 86% in *CrERF5*-silenced plants compared with the controls. In the *CrERF5*-silenced plants, the expression levels of two iridoid pathway genes (*GES* and *SLS1*), vindoline pathway gene *T16H1*, indole pathway gene *TDC*, and downstream MIA pathway genes (*STR*, *SGD, Redox1, SAT*, and *PRX1*) were significantly downregulated compared to the controls. But the expression levels of other genes did not significantly differ between the *CrERF5*-silenced plants and the controls (Figures 4A–C).

**FIGURE 5:**
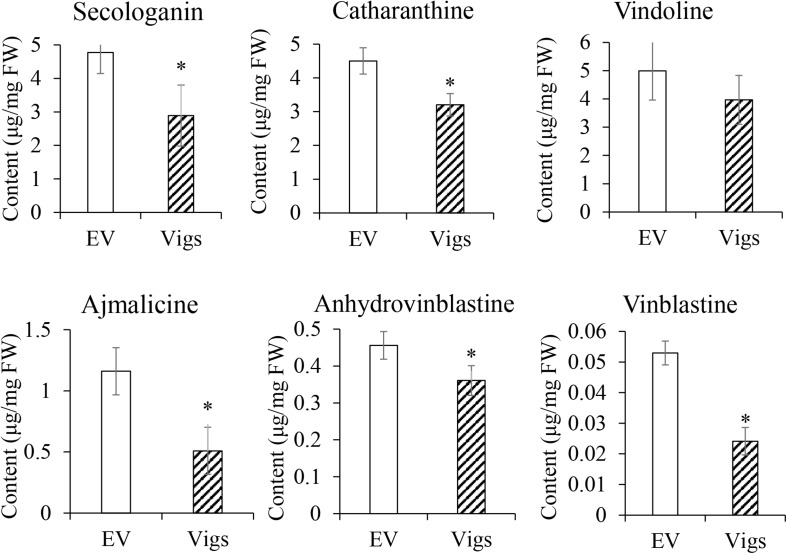
Contents of secologanin, ajmalicine, catharanthine, vindoline, anhydrovinblastine, and vinblastine in *CrERF5*-silencing *C. roseus* lines. The error bars represent the means ± SD from three biological replicates, and asterisks indicate statistically significant differences compared with the controls. ^*^P < 0.05.

Subsequently, the contents of five MIAs (catharanthine, vindoline, ajmalicine, anhydrovinblastine, and vinblastine) and one precursor secologanin were measured by UPLC-Q/TOF MS (Figure 5). The accumulation of secologanin, catharanthine, ajmalicine, anhydrovinblastine and vinblastine in the leaves of *CrERF5*-silenced plants significantly downregulated with a decrease of 39.4, 28.9, 56.1, 20.9, and 54.4%, respectively, compared to the controls. The levels of catharanthine and vindoline showed a slight decrease. These results are consistent with the changes of the expression levels of MIAs biosynthetic genes in *CrERF5*-silenced plants, which indicated that CrERF5 might function as a positive regulator to regulate MIAs biosynthesis.

### Transient Expression of CrERF5 Induces Expression of MIA Pathway Genes in *C. roseus* Flower Petals and Boosts MIAs Accumulation

**FIGURE 6:**
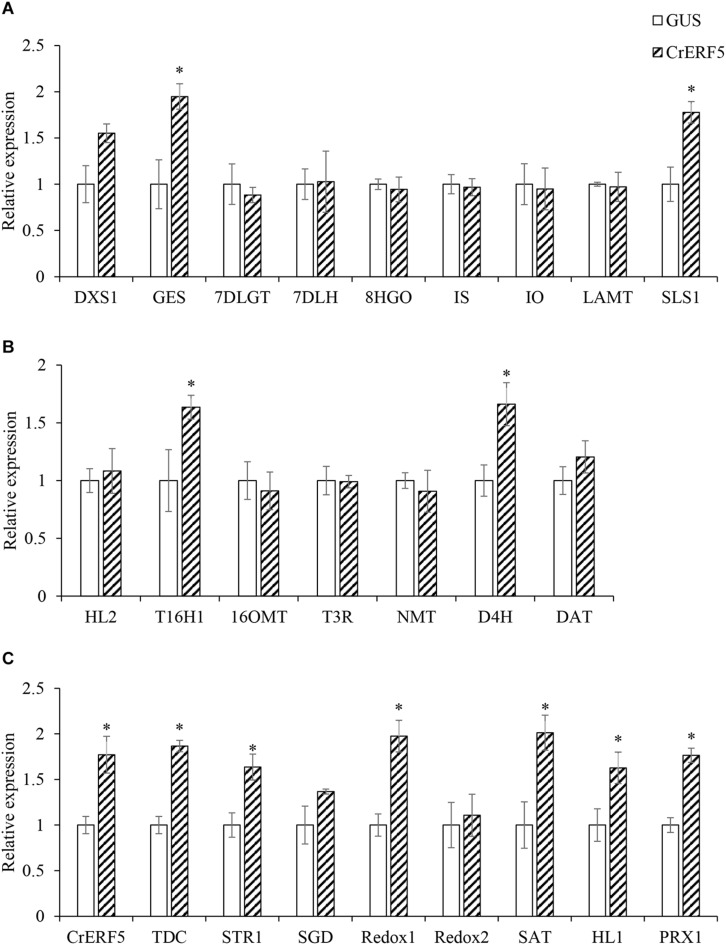
Expression of *CrERF5* and MIA pathway genes in *C. roseus* flower petals transiently overexpressing *CrERF5*. **(A)** Iridoid pathway genes; **(B)** Vindoline pathway genes; **(C)** Indole and MIA pathway genes. The error bars represent the means ± SD from three biological replicates, and asterisks indicate statistically significant differences compared with the controls. ^*^P < 0.05.

**FIGURE 7:**
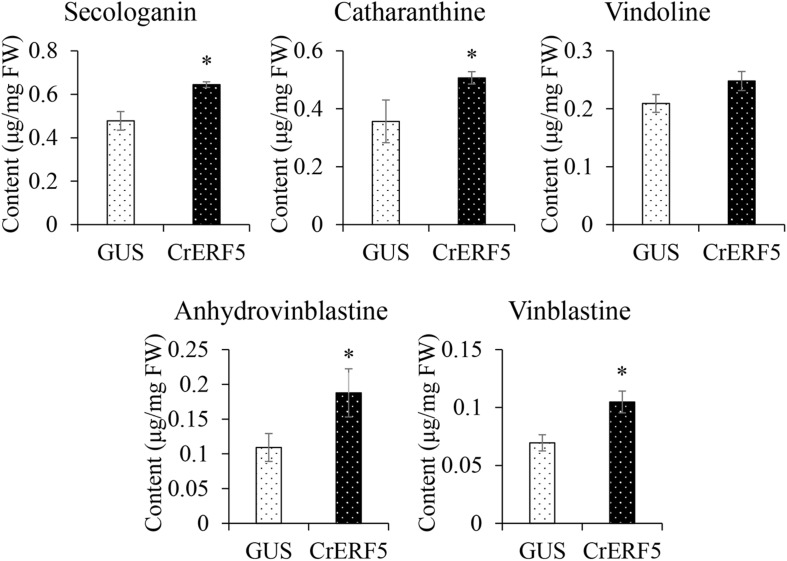
Contents of secologanin, ajmalicine, catharanthine, vindoline, anhydrovinblastine, and vinblastine in *C. roseus* flower petals transiently overexpressing *CrERF5*. The error bars represent the means ± SD from three biological replicates, and asterisks indicate statistically significant differences compared with the controls. ^*^P < 0.05.

To further confirm the function of CrERF5 in MIA biosynthesis, CrERF5 was transiently overexpressed in *C. roseus* flower petals. The results showed that overexpressing *CrERF5* strongly activated the expression of MIA biosynthetic genes in both upstream and downstream pathways (Figures 6A–C). The significant induction of iridoid pathway genes *GES* and *SLS1*, indole pathway gene TDC, MIA biosynthetic genes *STR1, Redox1, SAT, HL1*, and *CrPRX1*, and vindoline pathway genes *T16H1* and *D4H* was observed by transient CrERF5 overexpression. Next, the contents of five MIAs and one precursor were detected and analyzed as the previous part. Levels of the detectable MIAs and precursor were all significantly enhanced (Figure 7 and Supplementary Figure S5). Compared to the controls, the content of secologanin increased 34.9%, catharanthine increased 42.1%, anhydrovinblastine increased 71.9%, and vinblastine increased 50.7%. The results confirmed that CrERF5 plays a positive role in the regulation of the MIA pathway.

## Discussion

In *C. roseus*, the reported AP2/ERF transcription factors, ORCAs and CR1, are mainly induced by the plant hormone jasmonate (JA) to regulate the MIA biosynthesis (Van der Fits and Memelink, 2000, 2001). The CrMYC2 JA-signaling module upregulates ORCAs expression, in turn activates several genes (TDC, CPR, and STR) in the upstream MIA pathway, steering the steps leading to strictosidine. Although more TFs are reported to be involved in JA-mediated regulation of MIA biosynthesis, such as BIS1/2, RMT1, CrWRKY1, CrGBF1/2, and the ZCTs, the regulatory network of MIA biosynthesis in *C. roseus* organs remains not well understood yet. For example, expression of the vinblastine, catharanthine and vindoline pathway genes is not regulated by the JA-responsive CrMYC2/BIS/ORCA module (Schweizer et al., 2018). All above indicated that besides JA-inducible TFs, many other hormone-inducible regulatory TFs need to be discovered still. Here, we have demonstrated that CrERF5 acts as a new ethylene-JA-inducible transcription factor upregulating the MIA pathway leading to the bisindole alkaloids accumulation.

In *Arabidopsis*, AtERF5 functioned as an activator of GCC box-dependent transcription and upregulated the expression of peroxisome-associated gene PEX1 (Salt-induced) (Fujimoto et al., 2000; Charlton et al., 2005; Mito et al., 2011). SlERF5 is a transcription activator for genes involved in the responses to ABA, biotic and abiotic stress (Chuang et al., 2010). NtERF5-Overexpressing plants show enhanced resistance to TMV with reference to reduced size of local hypersensitive-response lesions and impaired systemic spread of the virus (Fischer and Dröge-Laser, 2004). In our transcriptome analysis of *C. roseus* tissues (flower, leaf, stem, and root), CrERF5 was co-expressed with SGD, ORCA3, and D4H, and the activation of TDC promoter by CrERF5 verified its involvement in MIA biosynthesis.

The expression pattern of CrERF5 in organs indicated that CrERF5 transcripts increased with leaf age. As reported, the anhydrovinblastine level increases with leaf age, in parallel with an increase of CrPrx1 expression level (Costa et al., 2008; Pan et al., 2016b). CrERF5 expression pattern consisted with the accumulation pattern of bisindole alkaloids, which give an explanation why CrERF5 had a positive effect on the anhydrovinblastine and vinblastine contents. And CrERF5 expression was induced by ethylene or JA treatment. Moreover, ethylene induction had a stronger effect on CrERF5 expression than JA induction, not like other reported AP2/ERF TFs that were mainly induced by JA signaling. Previous studies have showed that exogenous ethylene not only elevate the Cd resistance of *C. roseus* but also effectively influence MIAs biosynthesis in *C. roseus* (Chen et al., 2017). Significant accumulation of vinblastine was found in response to high concentration of ethylene and Cu, in which ERF and MPK formed a positive feedback loop connecting two pathways actively involved in *C. roseus* (Pan et al., 2014). Comprehensive profiling of the vinblastine biosynthesis in response to ethephon suggested that ERF could regulate and interact with CrPRX1 to play a key role in vinblastine accumulation and peroxidase activity (Wang X. et al., 2016). These previous researches supported our results that CrERF5, responding to both ethylene and JA signals, might be involved in the bisindole alkaloids accumulation.

**FIGURE 8:**
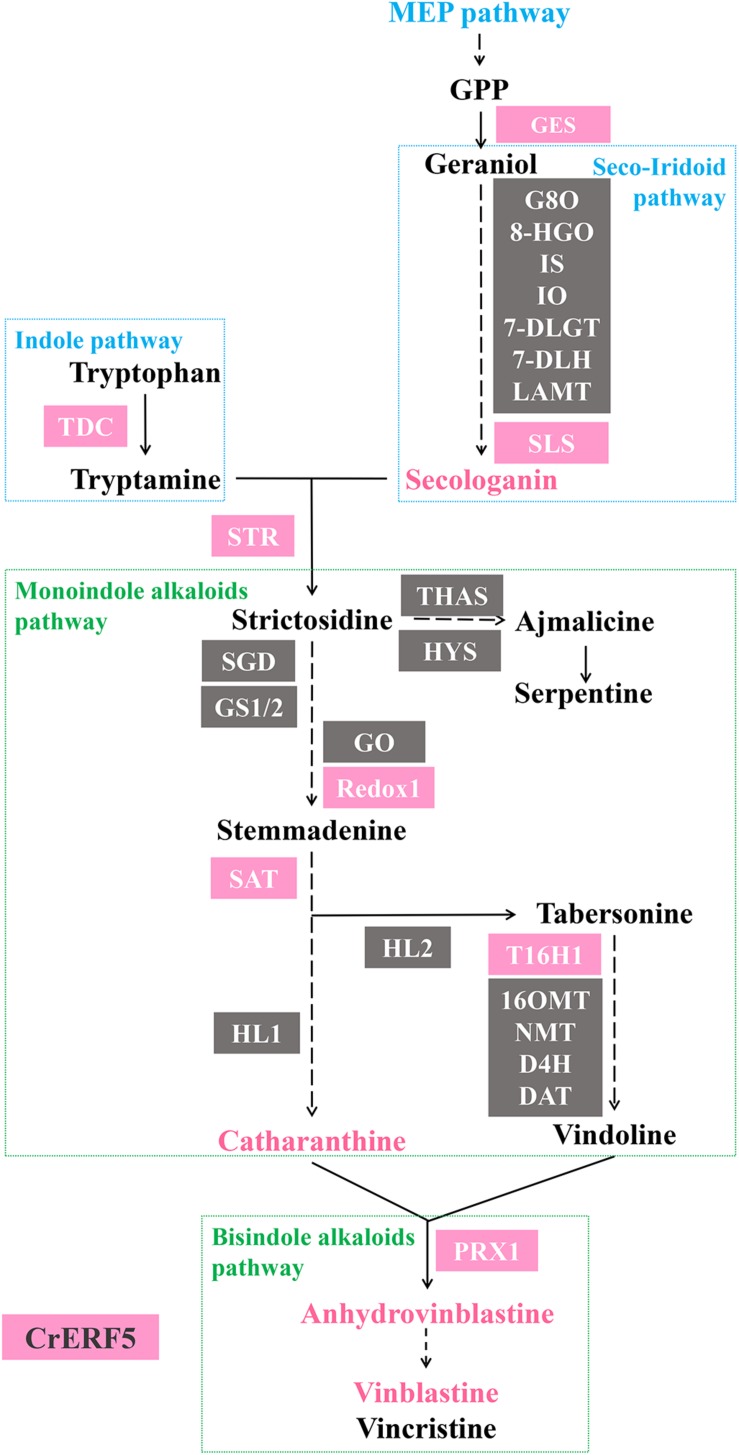
A scheme of CrERF5 regulation on MIA biosynthetic pathway. Genes regulated by CrERF5 are boxed in pink, respectively. Genes not regulated by CrERF5 are boxed in gray. MIAs upregulated by CrERF5 are in pink.

Further investigation via VIGS and transient petal expression showed that CrERF5 had a positive regulation on both upstream and downstream MIA pathways (Figure 8). The expression of indole pathway gene *TDC* was induced and activated by CrERF5, in turns which facilitated the boost of MIAs in the downstream pathway branches. CrERF5 upregulated the expression of GES and SLS and secologanin accumulation. Except *GES* and *SLS*, other genes in the iridoid pathway, like *7-DLGT, 7-DGT, IO, IS*, and *LAMT*, were not under the control of CrERF5. Moreover, *DXS1*, the first step enzyme gene of MEP pathway, was repressed by CrERF5 in Dual-LUC assay. Given that GES and SLS are the enzymes in the closer catalyzing steps to form secologanin than DXS1, CrERF5 repression on DXS1 might have little effect on the secologanin biosynthesis. Vindoline pathway genes were partly regulated by CrERF5. But both overexpressing and silencing CrERF5 had limited effect on vindoline accumulation, indicating that CrERF5 might not control vindoline pathway. It has been reported that light-induced vindoline biosynthesis is regulated by GATA and PIF transcription factors in *C. roseus* (Liu et al., 2019). But the genes in the steps from strictosidine to vinblastine, like *STR1, Redox1, SAT*, and *CrPRX1*, were upregulated in CrERF5-overexpressing petals and downregulated in CrERF5-silencing plants, leading to a significant increase of the catharanthine, anhydrovinblastine and vinblastine accumulation. These results inferred that CrERF5 played a multiple positive role in regulating the MIA pathways, especially leading to the bisindole alkaloids biosynthesis.

## Conclusion

Our study found an important ethylene-JA-induced regulator CrERF5 that upregulates both the upstream and downstream MIA pathway branches, which could be used as a potent powerful tool to boost the high-value bisindole alkaloids production in the future. Combined with all these results, it indicated that in *C. roseus* ethylene signaling pathway might play an important role in transcriptional regulation on MIAs biosynthesis, especially bisindole alkaloids, which offered a new view to understand the complex molecular mechanism of MIAs biosynthesis.

## Accession Numbers

Gene sequences have been deposited in the GeneBank database under the accession numbers CrERF5 (MK862158), ORCA2 (CAB93940.1), ORCA3 (ABW77571.1), ORCA4 (KR703577), ORCA5 (KR703578), AtERF1 (O80337), AtERF2 (O80338), AtERF5 (O80341), AtERF13 (NM_130048.3), AaERF1 (AEQ93554.1), AaERF2 (AEQ93555.1), AaERF3 (JN695782.1), AaORA (JQ797708), NtERF2 (NP_001311965.1), NtERF3 (NM_001325724.1), NtERF5 (AY655738.1), SlERF1 (NM_001247912.2), SlERF2 (NM_001247379.2), SlERF3 (NM_001246867), SlERF4 (NM_001247384.2), SlERF5 (AAS72389), SlERF6 (JN616265.1), and OpERF5 (XP_009764685.1).

## Data Availability

Publicly available datasets were analyzed in this study. This data can be accessed from: https://www.ncbi.nlm.nih.gov.

## Author Contributions

QP made her contributions to the concept or the design of the work, data analysis, and manuscript writing. CW made her contributions in CrERF5 gene cloning and VIGS experiments. ZX contributed to the Dual Luciferase assays and the results analysis. HW contributed to the metabolite analysis by UPLC-Q/TOF MS. QS contributed to the analysis of RNA-seq data. XF contributed to the subcellular localization of CrERF5. BP contributed to the expression profile of CrERF5 in *C. roseus* tissues and under hormone treatments. YM contributed to the transient overexpression of CrERF5 in *C. roseus* flowers. XS assisted to the data collection and analysis. KT contributed to the experiment design and manuscript correction.

## Conflict of Interest Statement

The authors declare that the research was conducted in the absence of any commercial or financial relationships that could be construed as a potential conflict of interest.

## Supplementary Material

FIGURE S1 Monoterpenoid indole alkaloid (MIA) component analysis in *C. roseus* by UPLC-Q/TOF MS. **(A)** Chromatogram spectra of MIAs. **(B)** Mass spectra of MIAs and precursors.

FIGURE S2 MS/MS spectra of MIAs and precursor in petals and leaves. **(A)** MS/MS spectra of ajmalicine in flower, leaf, and standard; **(B)** MS/MS spectra of secologanin in flower, leaf, and standard; **(C)** MS/MS spectra of vindoline in flower, leaf, and standard; **(D)** MS/MS spectra of catharanthine in flower, leaf, and standard; **(E)** MS/MS spectra of anhydrovinblastine in flower, leaf, and standard; **(F)** MS/MS spectra of vinblastine in flower, leaf, and standard.

FIGURE S3 Amino acid sequence alignments of the CrERF5 protein with ERF5 from other species. Identical amino acid residues are highlighted in black, and the upper line indicates the AP2/ERF domain.

FIGURE S4 Subcellular localization of 35S:CrERF5-YFP (top) and 35S:YFP (below) in *Nicotiana benthamiana* leaf epidermal cells. Yellow, yellow fluorescent protein (YFP); blue, DAPI. Bars, 25 μm.

FIGURE S5 The contents of ajmalicine/geissoschizine in *C. roseus* flower petals transiently overexpressing *CrERF5*. The error bars represent the means ± SD from three biological replicates, and asterisks indicate statistically significant differences compared with the controls. ^*^*P* < 0.05.

TABLE S1 Polymerase chain reaction (PCR) primers used in this study.

TABLE S2 Fragments per kilobase of exon per million fragments mapped (FPKM) values of *C. roseus* AP2/ERF TFs, MIA pathway genes in flower, leaf, stem, and root. To tackle multiple contigs for certain genes, the contig with the highest FPKM was selected for the HCL analysis.
