# Supplementary figures and images for: CrERF5, an AP2/ERF Transcription Factor, Positively Regulates the Biosynthesis of Bisindole Alkaloids and Their Precursors in Catharanthus roseus
Source: Front Plant Sci. 2019 Jul 18;10:931. doi: 10.3389/fpls.2019.00931 (PMC6657538; doi:10.3389/fpls.2019.00931)

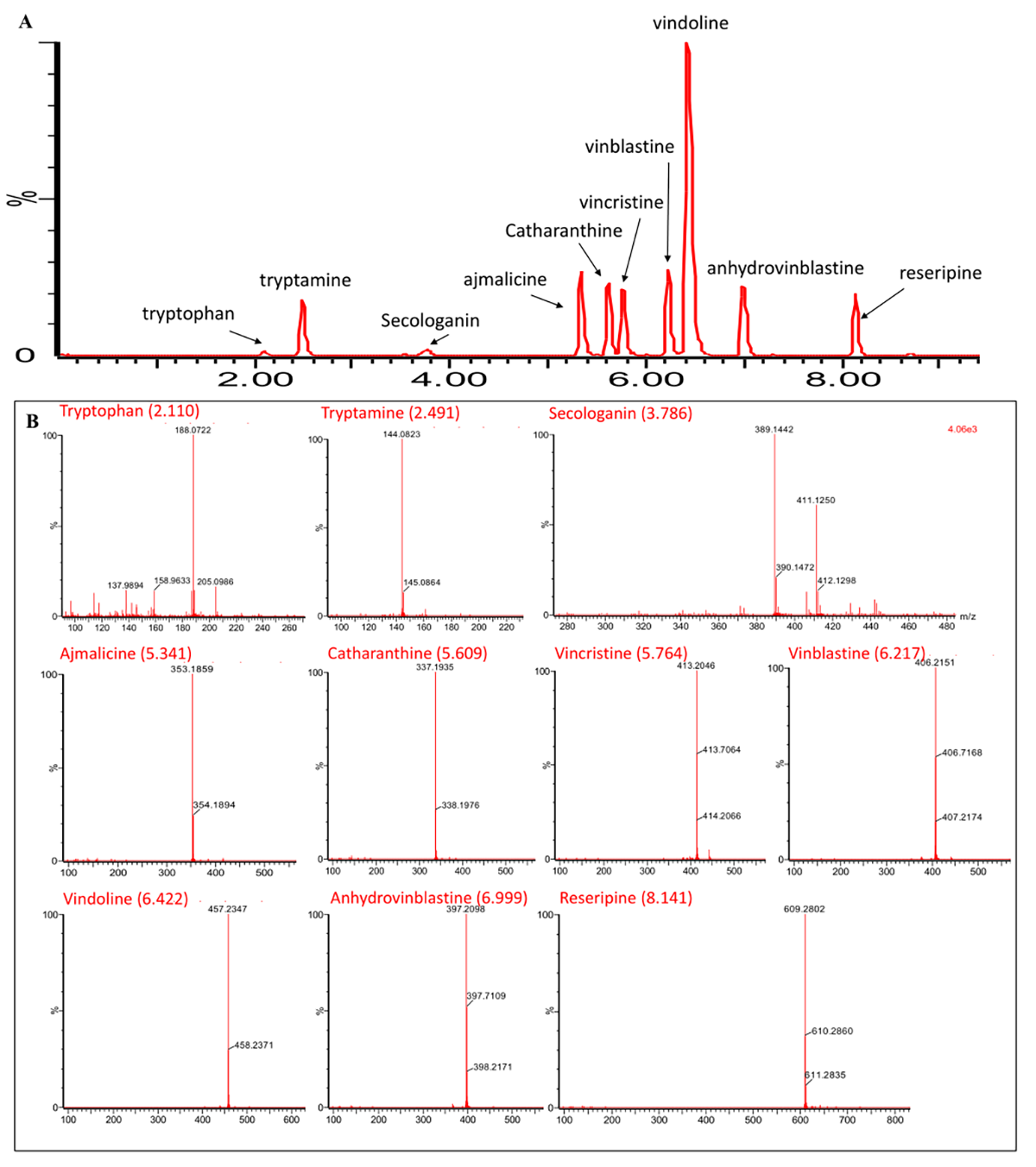

Supplement: FIGURE S1 — Monoterpenoid indole alkaloid (MIA) component analysis in C. roseus by UPLC-Q/TOF MS. (A) Chromatogram spectra of MIAs. (B) Mass spectra of MIAs and precursors. [file Image_1.TIF]

**A****Flower**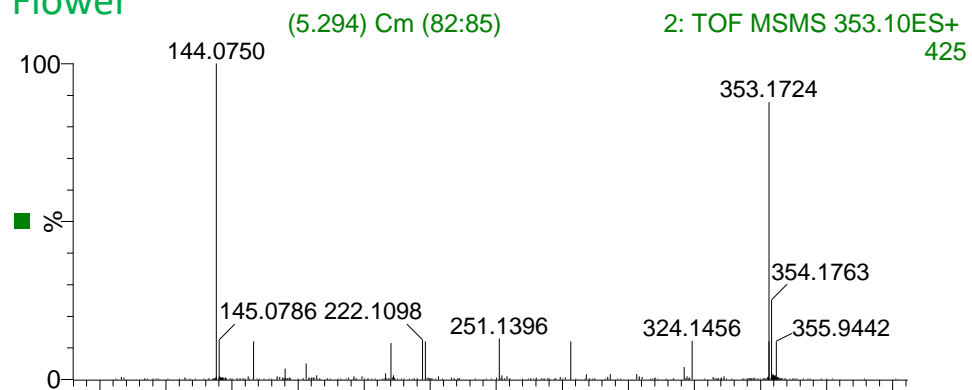**Leaf**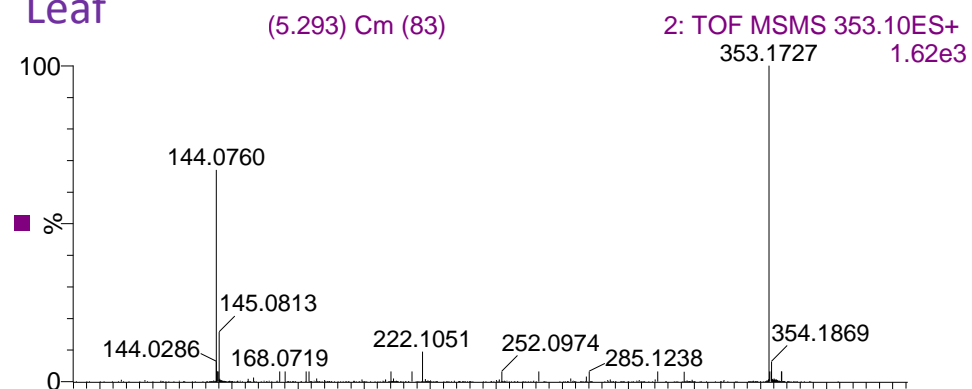**Standard**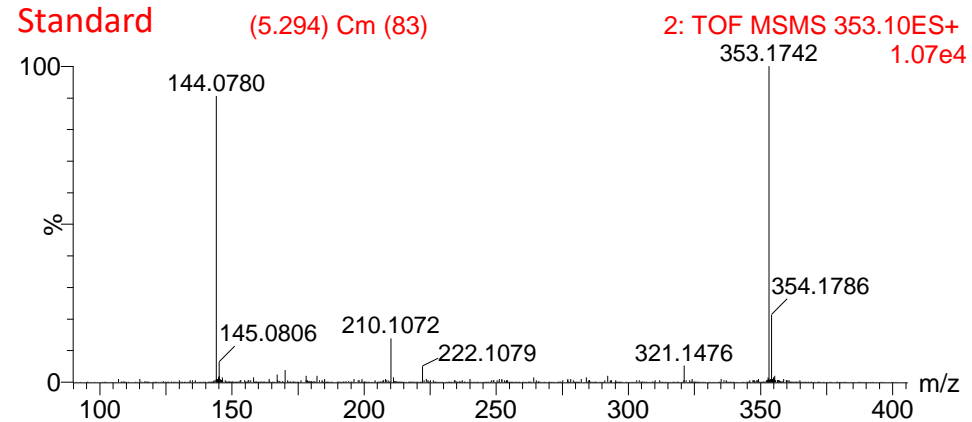**B****Leaf**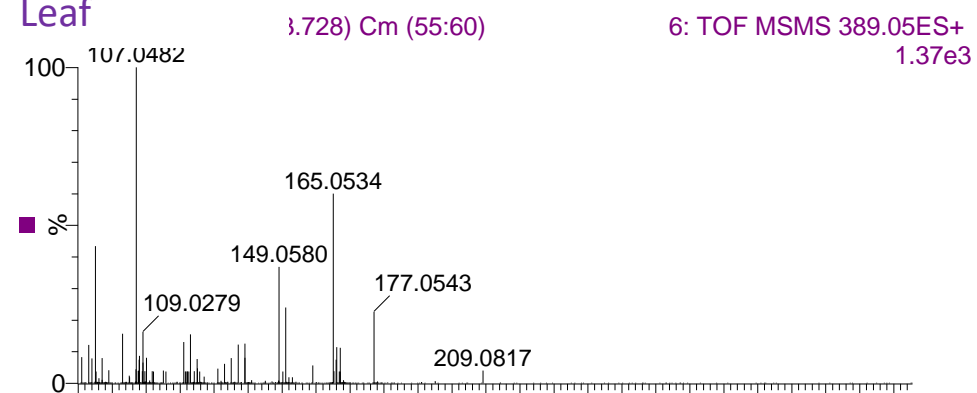**Flower**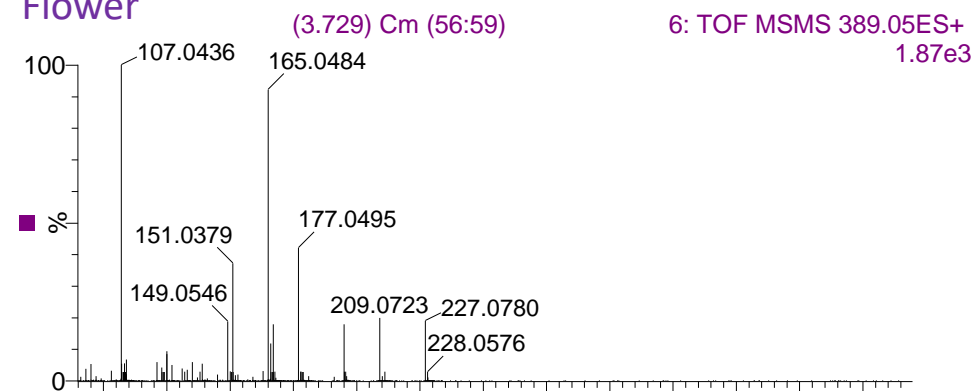**Standard**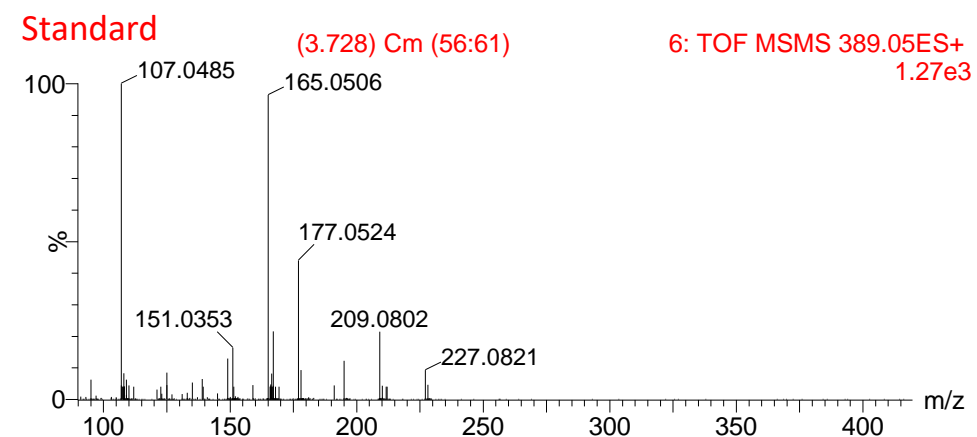

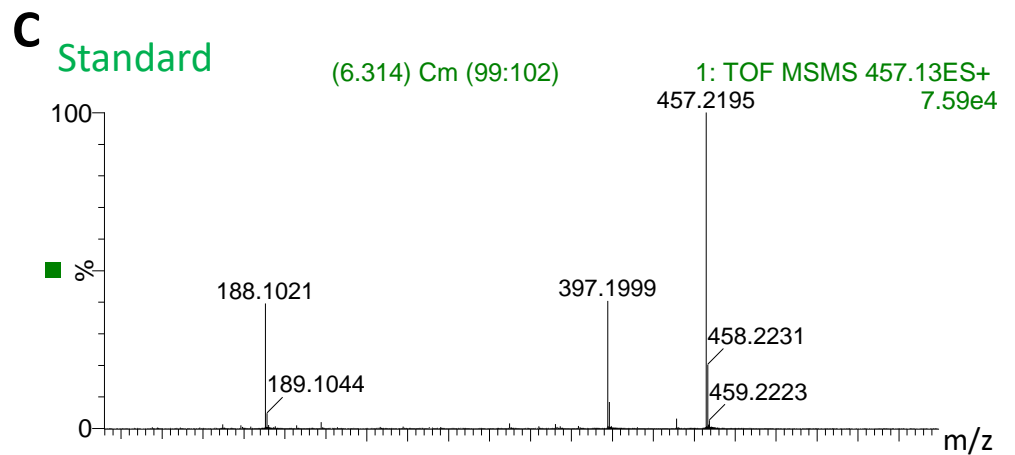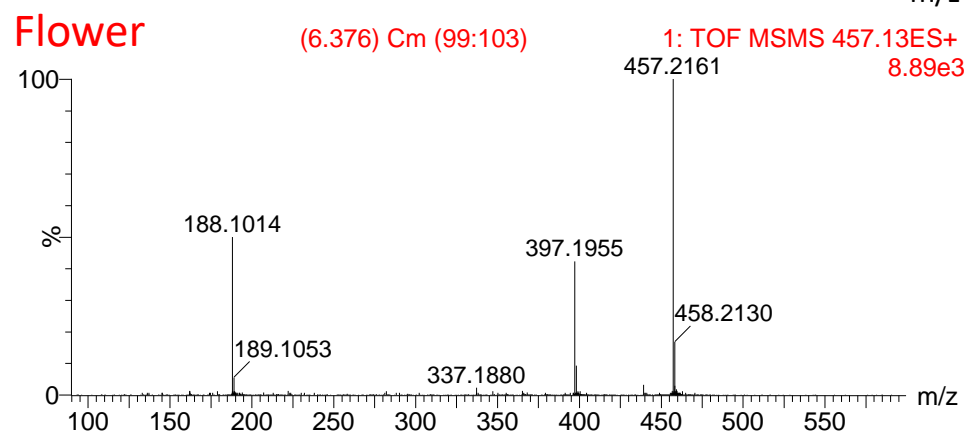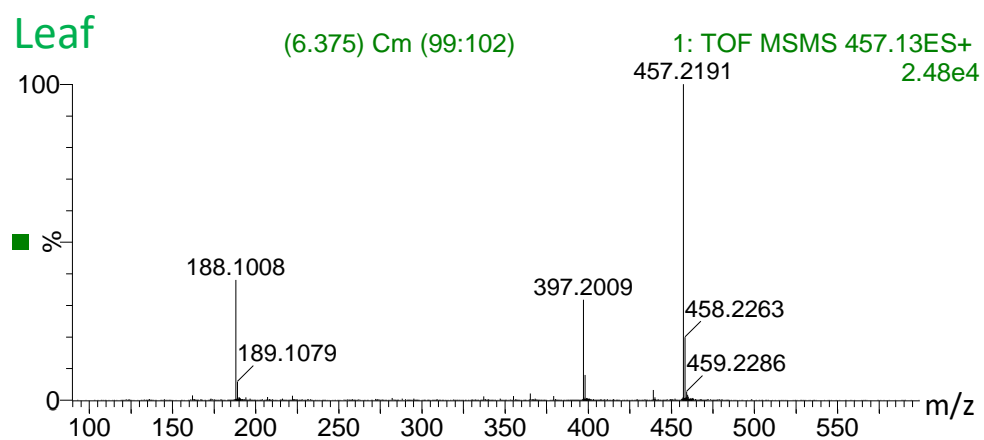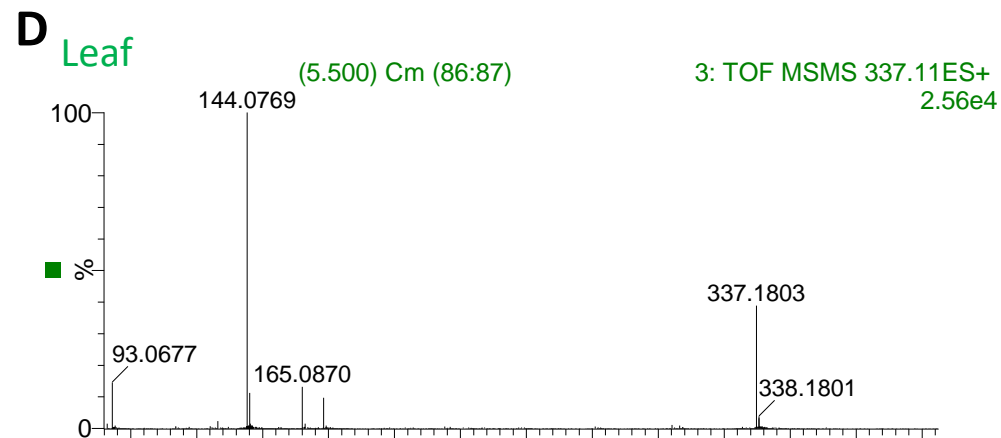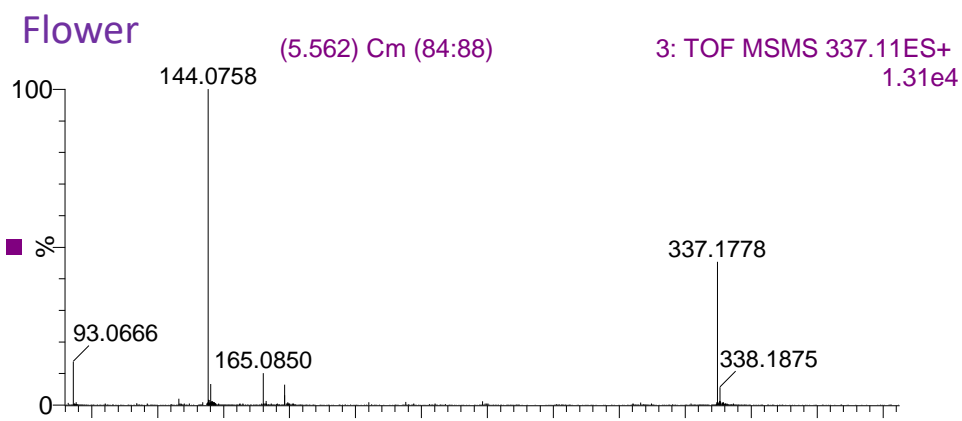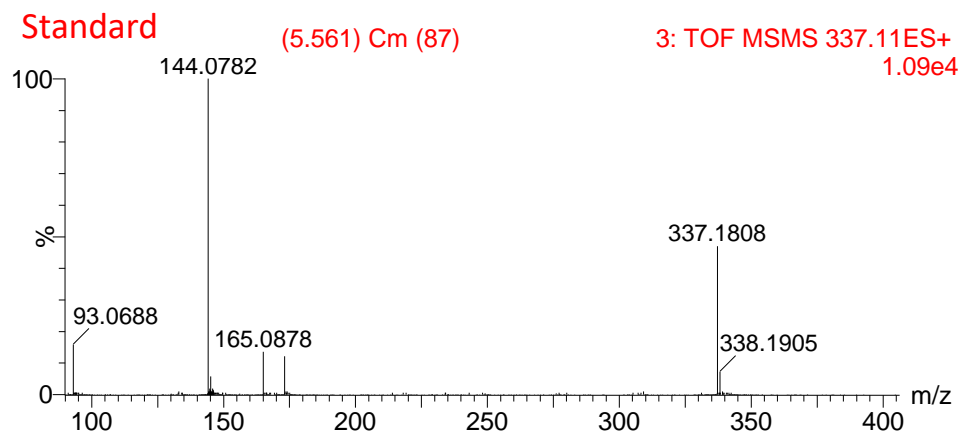

**E**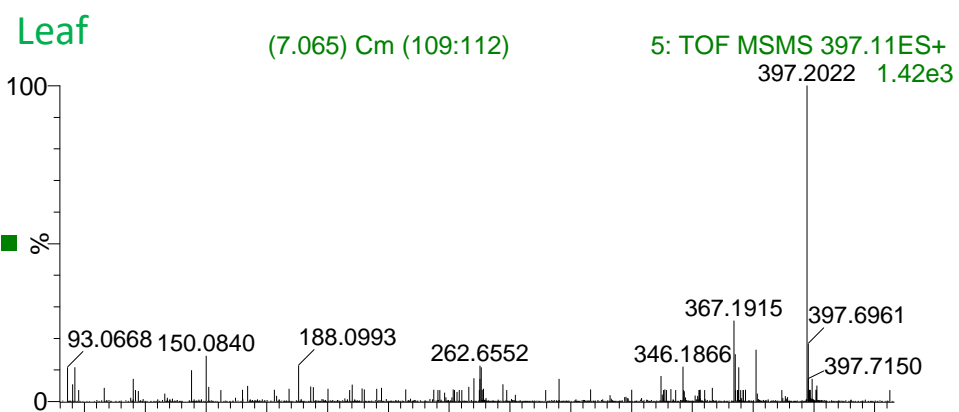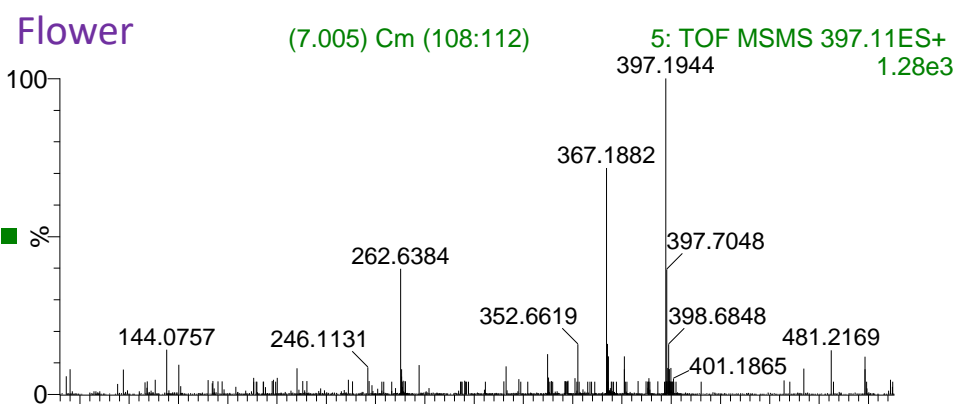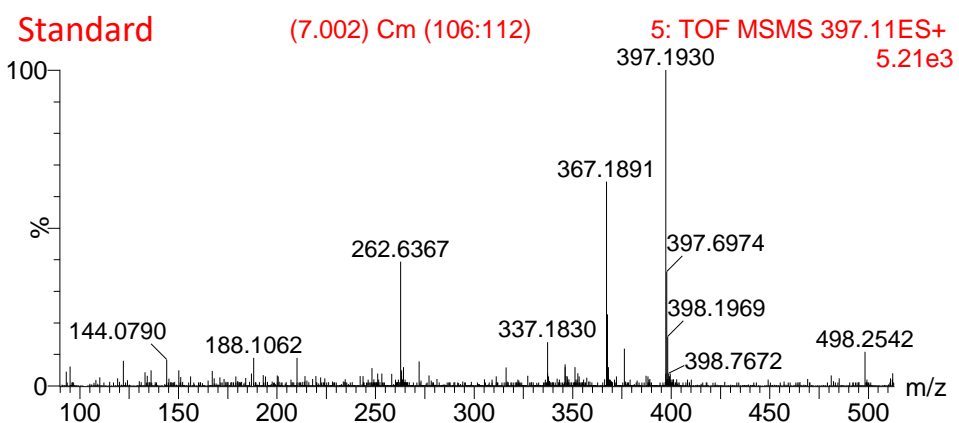**F**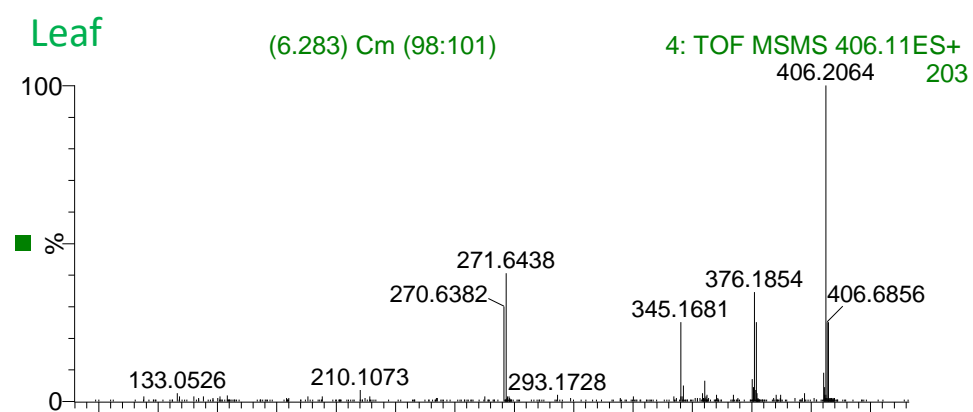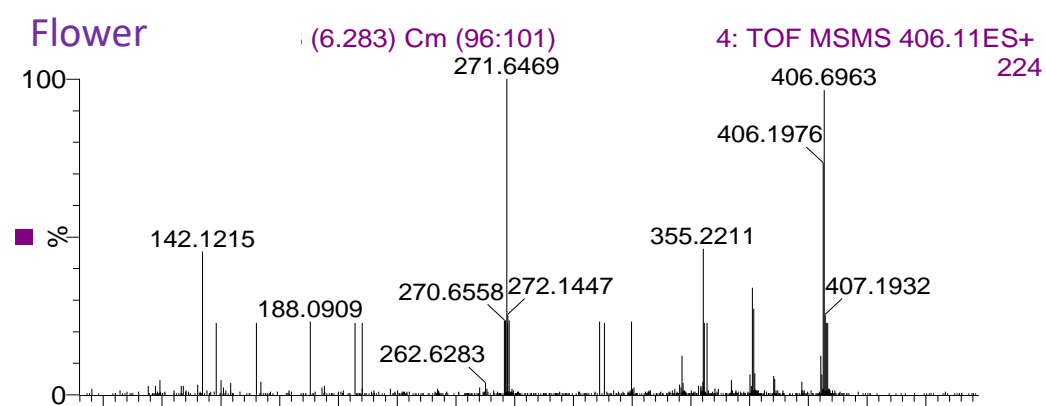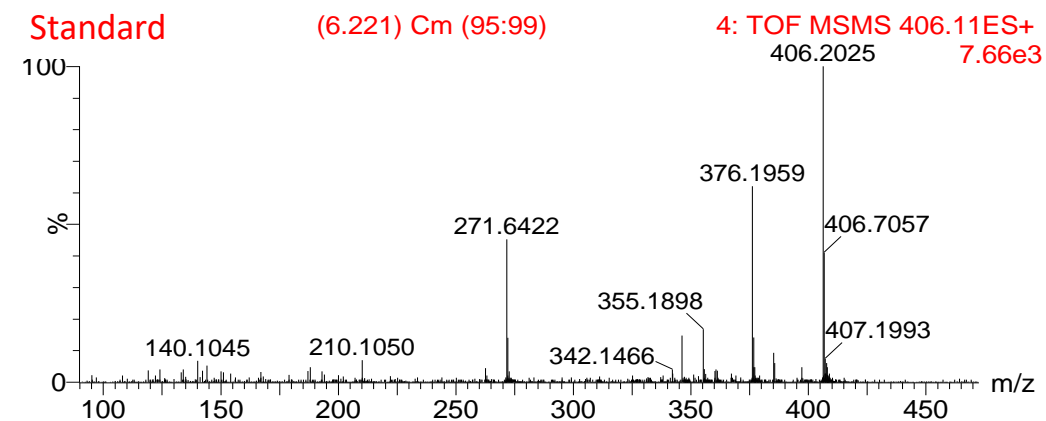

Supplement: FIGURE S2 — MS/MS spectra of MIAs and precursor in petals and leaves. (A) MS/MS spectra of ajmalicine in flower, leaf, and standard; (B) MS/MS spectra of secologanin in flower, leaf, and standard; (C) MS/MS spectra of vindoline in flower, leaf, and standard; (D) MS/MS spectra of catharanthine in flower, leaf, and standard; (E) MS/MS spectra of anhydrovinblastine in flower, leaf, and standard; (F) MS/MS spectra of vinblastine in flower, leaf, and standard. [file Image_2.pdf]

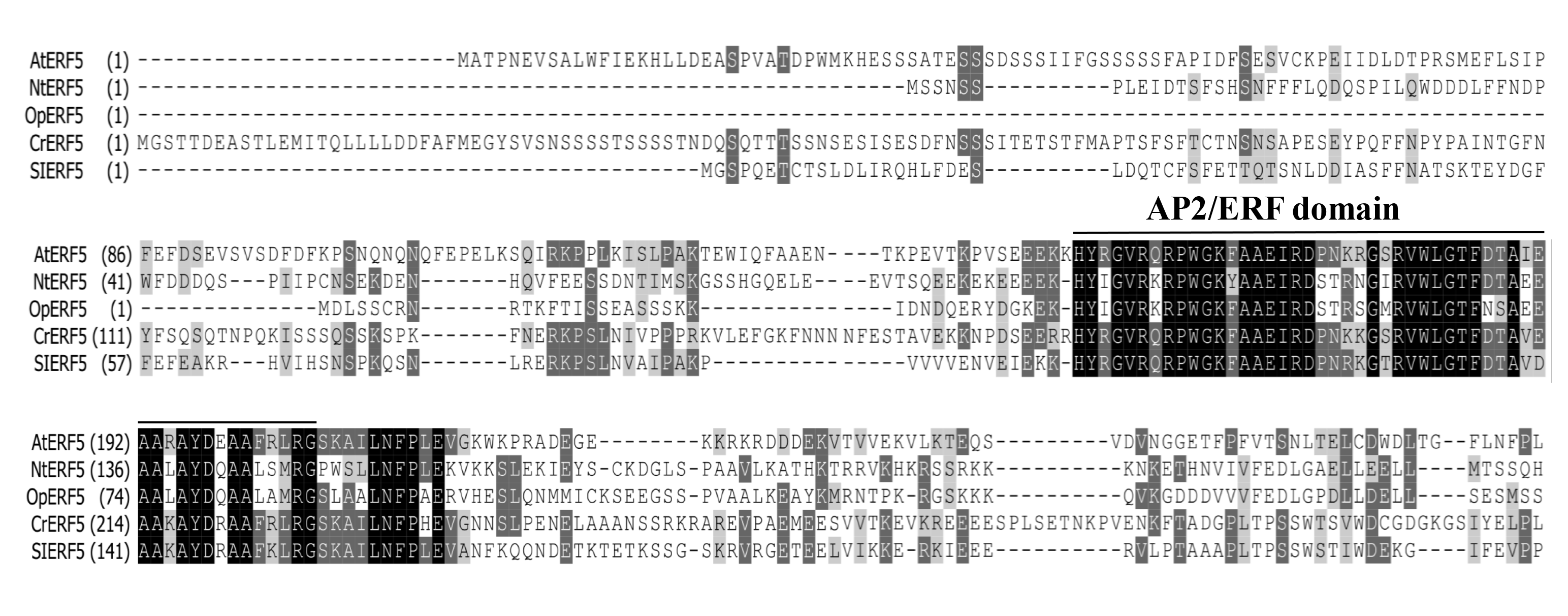

Supplement: FIGURE S3 — Amino acid sequence alignments of the CrERF5 protein with ERF5 from other species. Identical amino acid residues are highlighted in black, and the upper line indicates the AP2/ERF domain. [file Image_3.tif]

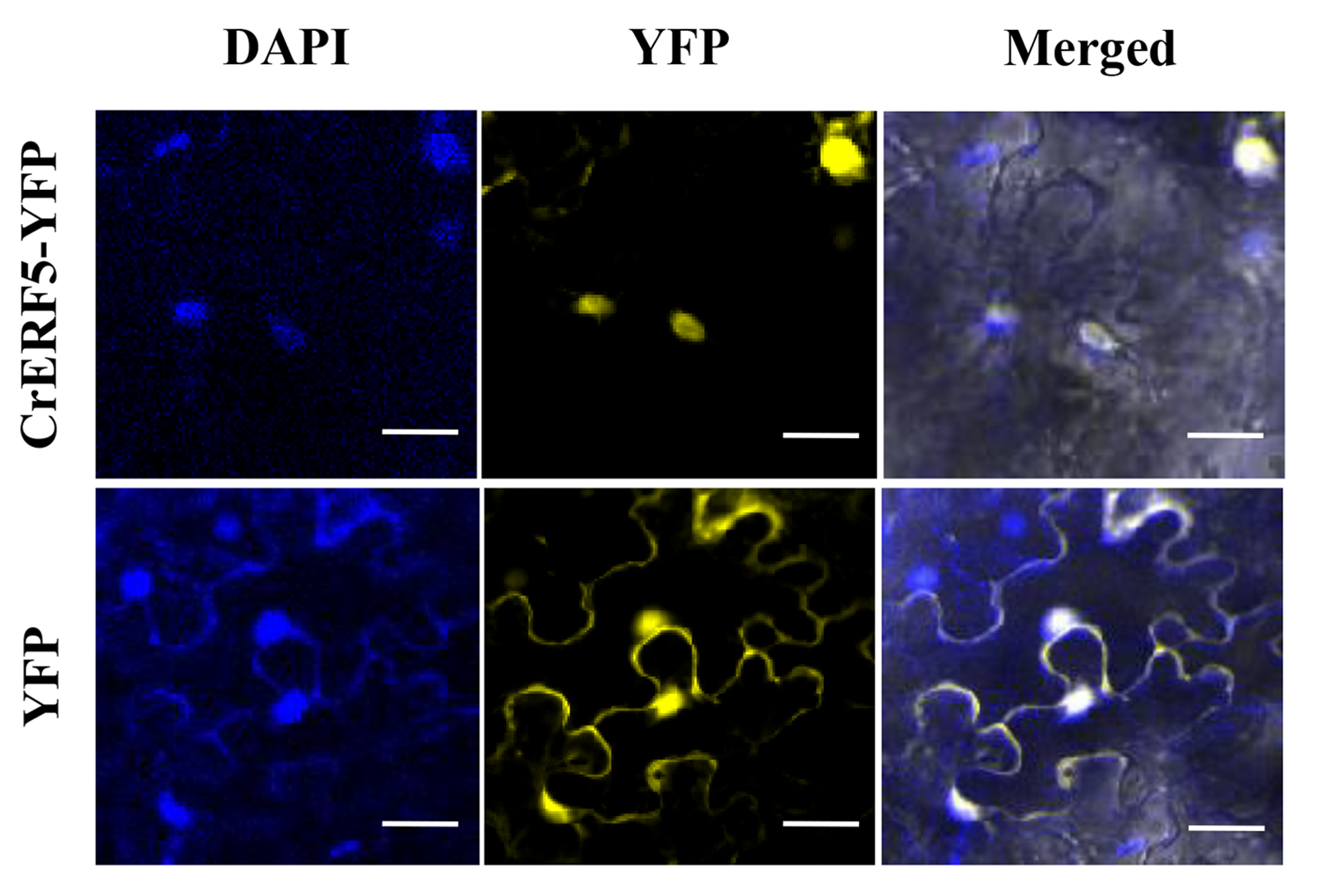

Supplement: FIGURE S4 — Subcellular localization of 35S:CrERF5-YFP (top) and 35S:YFP (below) in Nicotiana benthamiana leaf epidermal cells. Yellow, yellow fluorescent protein (YFP); blue, DAPI. Bars, 25 μm. [file Image_4.tif]

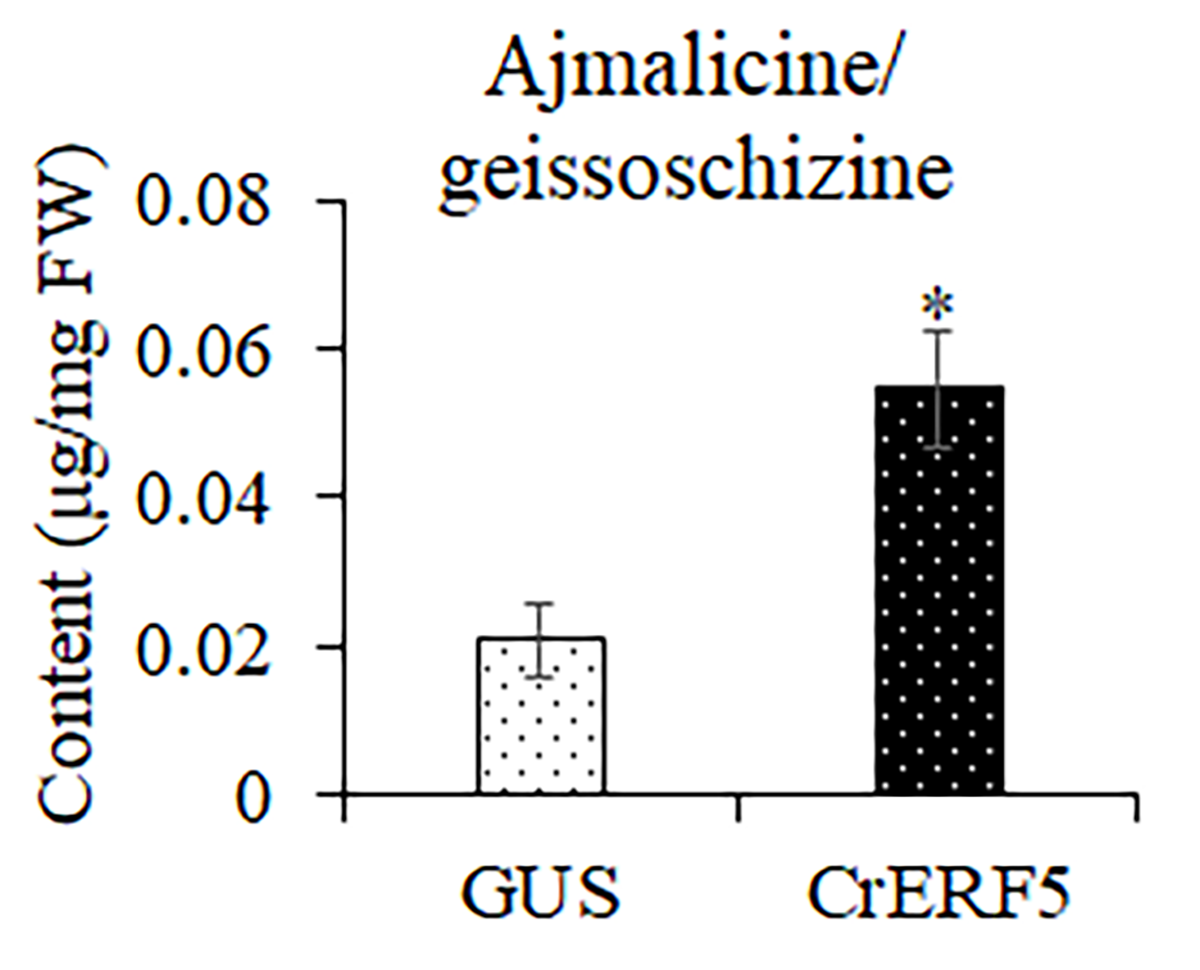

Supplement: FIGURE S5 — The contents of ajmalicine/geissoschizine in C. roseus flower petals transiently overexpressing CrERF5. The error bars represent the means ± SD from three biological replicates, and asterisks indicate statistically significant differences compared with the controls. *P < 0.05. [file Image_5.tif]
