# Supplementary material for: CrERF5, an AP2/ERF Transcription Factor, Positively Regulates the Biosynthesis of Bisindole Alkaloids and Their Precursors in Catharanthus roseus
Source: Front Plant Sci. 2019 Jul 18;10:931. doi: 10.3389/fpls.2019.00931 (PMC6657538; doi:10.3389/fpls.2019.00931)
Supplement: TABLE S1 — Polymerase chain reaction (PCR) primers used in this study. [file Table_1.DOCX]

Supplementary Table 1 Polymerase chain reaction (PCR) primers used in this study.

| **Primer name** | **Primer sequence (5' to 3')** | **Purpose** |
| --- | --- | --- |
| CrERF5-PLB-F | CGACTAGTATGGGATCAACAACAGATGAAG | Target fragment clone |
| CrERF5-PLB-R | GGGTAACCCTCATATGACCATTAATTCAG | Target fragment clone |
| CrERF5-PHB-YFP-F | TCTAAGCTTGGATCCATGGGATCAACAACAGATG | Target fragment clone |
| CrERF5-PHB-YFP-R | GCTCACCATACTAGTTATGACCATTAATTCAGAAG | Target fragment clone |
| CrERF5-vigs-F | GAAGGAGCCCTTCACCGGATCCAAAGGGCTAGAGAAGTAC | Target fragment clone |
| CrERF5-vigs-R | CGGGTTTGAGCTCAAAGAATTCTAGGGCTCAGAATCGTCT | Target fragment clone |
| DXS1-promoter-F | TTGCGGCCGCAACGTATTATGCCCCTACAT | Target fragment clone |
| DXS1-promoter-R | CCCATGGGATGGAGATTTTGGATTGGAGGT | Target fragment clone |
| TDC-promoter-F | GCGGCCGCATGGCTGCTAATGTATTAAT | Target fragment clone |
| TDC-promoter-R | TCTAGAGGTGTAGGTGTATGTGTAGAG | Target fragment clone |
| SGD-promoter-F | TTGCGGCCGCAAACCGTGGAGGTTCATTAT | Target fragment clone |
| SGD-promoter-R | CCCATGGGGACTGATCATCTTTAGATCCCAT | Target fragment clone |
| CrPRX1-promoter-F | TTGCGGCCGCAATCGACCAAGGAAAGCTT | Target fragment clone |
| CrPRX1-promoter-R | CCCATGGGAGGAAGGGAGGAAAAAACAAC | Target fragment clone |
| TDC-F | ATCCGATCAAACCCATACCA | Target gene in qRT-PCR |
| TDC-R | CGTCATCCTCGACCATTTTT | Target gene in qRT-PCR |
| SLS-F | GTTCCTTCTCACCGGAGTTG | Target gene in qRT-PCR |
| SLS-R | CCCATTTGGTCAACATGTCA | Target gene in qRT-PCR |
| STR-F | ACCATTGTGTGGGAGGACAT | Target gene in qRT-PCR |
| STR-R | ATTTGAATGGCACTCCTTGC | Target gene in qRT-PCR |
| SGD-F | GGAGGCTTCTTGAGTGATCG | Target gene in qRT-PCR |
| SGD-R | GCAAATTCACCAGTGGCATA | Target gene in qRT-PCR |
| CrPRX1-F | GCGATTCATCAGTGCTGCTGGTGGGA | Target gene in qRT-PCR |
| CrPRX1-R | GTGGAAGGTTTGCTATTGTGTCTGCC | Target gene in qRT-PCR |
| DXS1-F | TCGCTGCAGAACTTAGAGCA | Target gene in qRT-PCR |
| DXS1-R | GCCAACATCCCAAATGATTC | Target gene in qRT-PCR |
| DAT-F | CTTCTTCTCATCACGTACCAACTC | Target gene in qRT-PCR |
| DAT-R | ATACCAAACTCAACGGCCTTAG | Target gene in qRT-PCR |
| CrERF5-F | CTCCATGGTTAAGGGGAGAGAT | Target gene in qRT-PCR |
| CrERF5-R | ATGTACATTGGCCAAACACGGC | Target gene in qRT-PCR |
| 7DLGT-F | CCACCACAAGACCTGAAGAAAT | Target gene in qRT-PCR |
| 7DLGT-R | CTAGTCTGAGCATGGAGTTCACA | Target gene in qRT-PCR |
| 7DLH-F | CCAGGCAAGGATTTCATTATTC | Target gene in qRT-PCR |
| 7DLH-R | TTCAGTCCAAAGTCAGGCAAG | Target gene in qRT-PCR |
| 8HGO-F | GAGATAGATCATTCATTCAAATG | Target gene in qRT-PCR |
| 8HGO-R | CTCATCTCATTTCTCATTTCTC | Target gene in qRT-PCR |
| GES-F | GCTTTGTTTTTCACACCTTGT | Target gene in qRT-PCR |
| GES-R | CTTAGCACATTTTACTCTCTC | Target gene in qRT-PCR |
| IO-F | CCGGTTTTCTTCTCCTCCTTAT | Target gene in qRT-PCR |
| IO-R | CCGTATTTGGACTTGAGCTTGT | Target gene in qRT-PCR |
| IS-F | TCTTGGGTTTTAGGAATTCGATGA | Target gene in qRT-PCR |
| IS-R | AAACCAAACCCAAAGCAGAAAA | Target gene in qRT-PCR |
| LAMT-F | CACTTCTTCATCTCTCTCTTC | Target gene in qRT-PCR |
| LAMT-R | CAATGGAATCAATTGTGGCAAC | Target gene in qRT-PCR |
| T16H1-F | GCCCAAAACAGCCAATATTCAAACC | Target gene in qRT-PCR |
| T16H1-R | ATGTGATGAGTATGGCCACCGC | Target gene in qRT-PCR |
| 16OMT-F | AATGGGCATTTCTCTTTAAGGA | Target gene in qRT-PCR |
| 16OMT-R | CCGTAATACAAATTGGGTACAA | Target gene in qRT-PCR |
| T3R-F | GAAGGGCTACAGGGGAACAC | Target gene in qRT-PCR |
| T3R-R | CACCCACAATTTCATGCCCG | Target gene in qRT-PCR |
| NMT-F | TTCGTGAGATGGTTCGGGTG | Target gene in qRT-PCR |
| NMT-R | CGGCGCCGTCACATATTTTT | Target gene in qRT-PCR |
| D4H-F | ATAGTTAATCATGGGATTCCACAAGATGTT | Target gene in qRT-PCR |
| D4H-R | GTTCATGAAACTTACGAACTCCATCTAC | Target gene in qRT-PCR |
| Rodex1-F | GAAGTGACGGAAGTGGGGAACAAA | Target gene in qRT-PCR |
| Rodex1-R | TCGCATTCGCCACATGAGTCAA | Target gene in qRT-PCR |
| Rodex2-F | TCGCTTGGGGAAGTAATGCTGT | Target gene in qRT-PCR |
| Rodex2-R | TGAGACTTGCTCCTTGCTCGTA | Target gene in qRT-PCR |
| SAT-F | GGATGGGGAAAGCCTGTTTCTGTT | Target gene in qRT-PCR |
| SAT-R | CTTCAGCCATGCTGATCCATGCTT | Target gene in qRT-PCR |
| HL1-F | TGGGGCTGGCTTTTGTCTAGAATC | Target gene in qRT-PCR |
| HL1-R | TAAGCTGCGGGTAAAAGGTGCTCT | Target gene in qRT-PCR |
| HL2-F | TGCTCCTGGTGGAAATGATAACCC | Target gene in qRT-PCR |
| HL2-R | AATCAGCAACCTCGAGCAACCA | Target gene in qRT-PCR |
| N2227-F | GGTTGCTCTTCATTACGGATTT | Endogenous reference gene |
| N2227-R | TGCAGCATAGTAATGGTTTTGC | Endogenous reference gene |
